# Enhanced oral bioavailability of vancomycin in rats treated with long-term parenteral nutrition

**DOI:** 10.1186/s40064-015-1228-8

**Published:** 2015-08-22

**Authors:** Keizo Fukushima, Akira Okada, Yoriko Hayashi, Hideki Ichikawa, Asako Nishimura, Nobuhito Shibata, Nobuyuki Sugioka

**Affiliations:** Department of Clinical Pharmacokinetics, Faculty of Pharmaceutical Sciences, Kobe Gakuin University, Chuo-ku, Kobe, 650-8586 Japan; Department of Physical Pharmacy, Faculty of Pharmaceutical Sciences, Kobe Gakuin University, Chuo-ku, Kobe, 650-8586 Japan; Department of Biopharmaceutics, Faculty of Pharmaceutical Science, Doshisha Women’s College of Liberal Arts, Kyotanabe, Kyoto 610-0395 Japan

**Keywords:** Vancomycin, Absorption, Bioavailability, Pharmacokinetics, Parenteral nutrition, Intestinal atrophy

## Abstract

Long-term parenteral nutrition (PN) can induce intestinal atrophy, leading to a loss of epithelial integrity in the small intestines. This change may alter the intestinal permeability of vancomycin (VCM), a non-absorbable antibiotic. The aim of the present study was to investigate the effect of PN on the pharmacokinetics of VCM in rats. VCM was intravenously (5 mg/kg) or intraduodenally (20 mg/kg) administered to control and PN rats, which were prepared by administration of PN for 9 days. After intravenous administration, there were no significant differences in any of the VCM pharmacokinetic parameters between the control and PN rats. However, after intraduodenal administration, the maximum concentration and area under the plasma concentration–time curve of VCM in PN rats was approximately 2.4- and 2.6-fold higher, respectively, than in the control rats; the calculated bioavailability was approximately 0.5 and 1.3 % in control and PN rats, respectively. These results indicated that PN administration did not affect VCM disposition, but enhanced VCM absorption; however, the enhanced oral VCM bioavailability was statistically, not clinically, significant. Therefore, while long-term PN administration may play a role in the enhancement of VCM bioavailability, this effect may be negligible without any complications.

**Electronic supplementary material:**

The online version of this article (doi:10.1186/s40064-015-1228-8) contains supplementary material, which is available to authorized users.

## Background

Parenteral nutrition (PN) serves as a critical therapy for patients in conditions where oral ingestion is not adequate. In spite of its usefulness, there are also many possible complications, such as catheter-related bloodstream infection (Hvas et al. 2014), impaired glucose tolerance (Beltrand et al. 2007), and parenteral nutrition-associated liver disease (Nandivada et al. 2013). In addition, long-term PN administration can induce intestinal atrophy; the reduction of the mucosal barrier may mediate bacterial translocation (BT), in which the intestinal bacteria and/or their toxic products invade the bloodstream and induce production of inflammatory cytokines, subsequently leading to sepsis and multiple organ failure (Hatakeyama and Matsuda 2014; Li et al. 1999; Sun et al. 2006).

Selective decontamination of the digestive tract (SDD) prophylaxis of BT, which prevents the overgrowth of pathogens by enteral co-administration of non-absorbable antibiotics, such as colistin, tobramycin, amphotericin B, and vancomycin (VCM) (Benus et al. 2010; Cerdá et al. 2007; Roos et al. 2011) has been previously reported. It has been shown that any absorption of the non-absorbable antibiotic in the SDD regimen can be considered negligible under “normal conditions”; however, significant absorption of tobramycin was reported in critically ill patients (Oudemans-van Straaten et al. 2011), as was VCM in patients with chemotherapy-associated and/or Clostridium difficile colitis (Aradhyula et al. 2006; Bergeron and Boucher 1994). In addition, we previously reported the enhanced intestinal permeability of a hydrophilic dye, phenolsulfonphthalein, in PN-induced intestinal atrophy (Fukushima et al. 2015). On the basis of these findings, there is potential to enhance the absorption of typically non-absorbable antibiotics in patients with PN-induced intestinal atrophy. It is well known that trough concentrations of VCM are associated with its nephrotoxicity (Elyasi et al. 2012) and that the rapid infusion of VCM induces red man syndrome (Healy et al. 1990). Generally, the absorption via passive diffusion of a hydrophilic drug is rapid. Therefore, there are concerns about side effects caused by the systemic exposure after enteral administration of VCM.

The aim of the present study was to investigate the impact of PN-induced intestinal atrophy on the absorption of VCM and to assess its clinical significance. Based on the usage of VCM in SDD regimen, VCM was administered intraduodenally to rats administered PN, and the absorption and disposition of VCM were assessed with intravenous administration.

## Methods

### Materials and animals

An injectable formulation of vancomycin (VCM) hydrochloride was purchased from Shionogi Co., Ltd., (Osaka, Japan). PNTWIN^®^ No.3 and VITAJECT^®^ were purchased from Ajinomoto Pharmaceuticals Co., Ltd. (Tokyo, Japan) and Terumo Co., Ltd. (Tokyo, Japan), respectively. Ampicillin and bupivacaine were obtained from Meiji Seika Pharma Co., Ltd. (Tokyo, Japan) and AstraZeneca PLC (London, UK), respectively. All other reagents were of analytical grade and were used without further purification. Male Wistar rat (weighing 250 ± 10 g, 10-weeks old) were purchased from Nippon SLC Co., Ltd. (Hamamatsu, Japan). All animal experiments in the present study were approved by the Animal Experimentation Committee of Kobe Gakuin University (approval number: A14-31). Rats had free access to food and water and were acclimated in a temperature-controlled facility with a 12 h light/dark cycle for at least 3 days before use.

### Preparation of PN-administered rats and laboratory tests

**Scheme 1:**
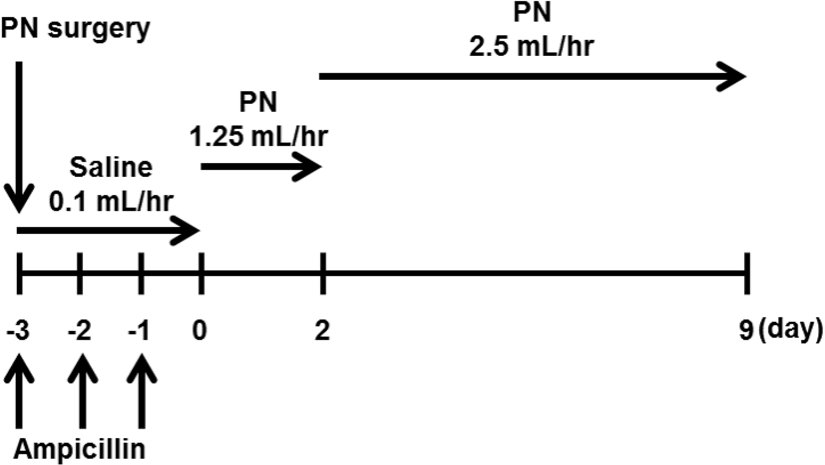
PN regimen

Parenteral nutrition-administered rats (PN rats) were prepared by the same method in our previous report (Fukushima et al. 2015) with only a change in the duration of PN administration; the preparation scheme of the PN rats are shown in Scheme 1. Briefly, 3 days before the start of PN administration, cardiac catheterization was performed via the right jugular vein with a polyurethane catheter (0.6-mm ID, 0.9-mm OD, Primetech Co., Tokyo, Japan) in rats anesthetized with sodium pentobarbital, and given ampicillin for prevention of infection and local bupivacaine for pain relief. The cannulated rats were housed individually in cages with free access to food and water, and received saline at a rate of 0.1 mL/h for 3 days via the catheter with a syringe pump (ISIS Co., Ltd., Osaka, Japan), and daily ampicillin (2 mg/kg, 2-min infusion) for recovery from surgery. For PN administration, infusion of PN solution was started at a rate of 1.25 mL/h for 2 days under fasting and water-deprived conditions; PN solution consisting of glucose, amino acids, electrolytes, and vitamins (approximately 0.97 kcal/mL) was prepared by mixing PNTWIN No. 3 with VITAJECT (Fukushima et al. 2015). Subsequently, PN administration was performed at a rate of 2.5 mL/h (viz., 60 mL/day, 58 kcal/day) for 7 days; likewise, the sham operated rats (control rats) underwent the same regimen with saline and allowed free access to food throughout the treatment. All rats were fasted overnight before the VCM pharmacokinetic study, and blood samples for laboratory testing were taken just before VCM administration; total protein (TP), serum albumin level (Alb), total cholesterol (T-Cho), triglyceride (TG), aspartate transaminase (AST), alanine transaminase (ALT), blood urea nitrogen (BUN), and serum creatinine (CRE) were measured by a commercial laboratory, Oriental Yeast Co., Ltd. (Tokyo, Japan).

### Intravenous and intraduodenal administration of VCM

The fasted control and PN rats were allocated to two groups (n = 4/group) based on the administration route: intravenous and intraduodenal VCM administration. In the intravenous administration study, rats were anesthetized with sodium pentobarbital (50 mg/kg), and the VCM saline solution (2.5 mg/mL) was bolus injected into the left femoral vein (5 mg/kg). Blood samples were taken 5, 15, 30, 45, 60, 90, 120, 180, 240, and 360 min after administration, and were centrifuged at 12,000 rpm for 15 min to collect the plasma fraction. In the intraduodenal administration study, the abdominal cavity was opened in anesthetized rats and the stomach was exposed. A small incision was made on the lesser curvature of the stomach, and the VCM saline solution (10 mg/mL) was administered through the incision and the pylorus into the duodenum (20 mg/kg) using a sterilized oral feeding needle. After administration, the incision was closed with a tissue adhesive and the pylorus was ligated. Blood samples were taken 5, 15, 30, 60, 90, 120, 180, 240, 300, and 360 min after administration, and plasma samples were collected by centrifugation. The collected plasma samples in both studies were immediately frozen at −80 °C until analysis.

### VCM assay

The VCM assay was performed by a previously reported liquid chromatography/tandem mass spectrometry (LC/MS/MS) method (Shibata et al. 2003) with some modifications; briefly, a 100 µL plasma sample was added to 60 µL of 30 % trifluoroacetic acid to precipitate protein. After vortexing and centrifugation at 12,000 rpm for 15 min, the supernatant was diluted in 340 µL of distilled water and was passed through a filter, and 10 µL of the filtrate was injected into a Quattro Ultima LC/MS/MS system with a 2690 Separation Module (Waters Co, MA, UK). VCM separation was performed with a QUICKSORB ODS column (i.d. 2.1 mm × 100 mm, 3 µm, Chemco Scientific Co., Ltd., Osaka, Japan), and the elution was carried out isocratically at a flow rate of 0.2 mL/min with the degassed mobile phase, acetonitrile: 0.1 % acetic acetate (2:8). Mass spectrometry was conducted with electrospray ionization in positive mode (ESI+) under the following conditions: source temperature, 130 °C; cone voltage, 35 V; capillary voltage, 4.0 kV. VCM intensity was monitored by multiple reaction monitoring (MRM) with 18 eV of collision energy for the VCM transition (725–144 *m*/*z*). VCM concentration was quantified by calculating peak area against calibrated samples. The lower limit of quantitation for VCM was <0.005 µg/mL.

### Pharmacokinetic analysis

A one- or two-compartment model with first-order absorption process was applied to the plasma concentration profiles of VCM; on the basis of the Akaike’s Information Criteria (AIC), the latter model was selected in the present study. All pharmacokinetic analyses were performed using WinNonlin^®^ Version 6.3 (Pharsight, Mountain View, CA). The structural parameters, including the absorption rate constant (*ka*), distribution volume of the central compartment (*V*_*c*_), distribution volume of the peripheral compartment (*V*_*p*_), total body clearance (*CL*_*tot*_), and distribution clearance (*CLD2*), and the secondary parameters including the maximum concentration (*C*_*max*_), time to maximum concentration (*T*_*max*_), distribution half-life (*t*_*1/2α*_), elimination half-life (*t*_*1/2β*_), and the area under the plasma concentration–time curve (*AUC*) were calculated. The bioavailability (*F*) was calculated using the following equation:

$$F = \left( {AUC_{{intraduodenal}} /Dose_{{intraduodenal}} } \right)/\left( {AUC_{{intravenous}} /Dose_{{intravenous}} } \right),$$

where *Dose* represents the administered dosage.

### Statistical analyses

All values are represented by the mean ± standard error (SE). Statistical differences of the means were assumed to be significant when *p* < 0.05 by the Mann–Whitney U test.

## Results

**Fig. 1:**
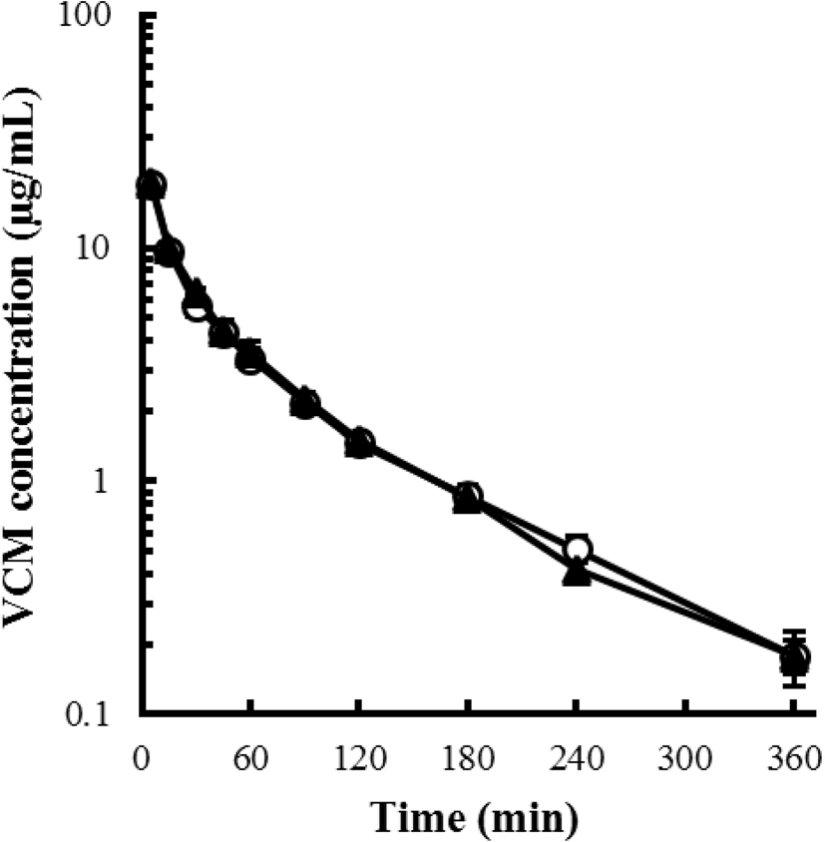
Plasma concentration profiles of VCM after intravenous administration (5 mg/kg) to (*open circles*) control and (*filled triangles*) PN rats. *Each symbol with a bar* represents the mean ± SE of 4 rats

**Fig. 2:**
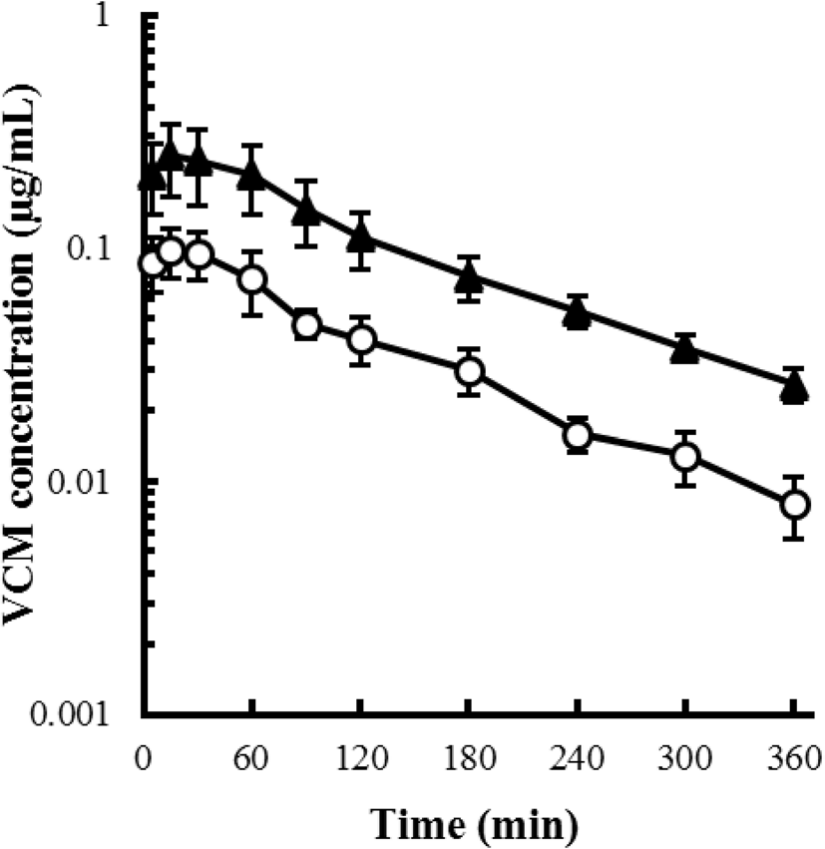
Plasma concentration profiles of VCM after intraduodenal administration (20 mg/kg) to (*open circles*) control and (*filled triangles*) PN rats. *Each symbol with a bar* represents the mean ± SE of 4 rats

Body weight and laboratory test results of the control and PN rats are shown in Additional file 1: Table S1. Body weight, TP, and Alb were slightly, but significantly, decreased in PN rats (approximately 88, 82 and 86 % of control, respectively), and TG levels in PN rats were markedly decreased, approximately 28 % of control. The plasma concentration profiles of VCM after intravenous and intraduodenal administration are shown in Figs. 1 and 2, respectively. After intravenous administration, the plasma concentration profiles of both control and PN rats were similar, and declined in a biphasic manner; after intraduodenal administration, the concentration of VCM in control and PN rats reached a peak within 30 min and then gradually decreased. The mean VCM concentration in PN rats was consistently higher than that in control rats after intraduodenal administration. The pharmacokinetic parameters of VCM are shown in Additional file 1: Table S2. After intravenous administration, there were no significant differences in any of the pharmacokinetic parameters between the control and PN rats; however, after intraduodenal administration, the *AUC* in PN rats was approximately 2.6-fold higher than that in control rats, with no other significant differences in the other pharmacokinetic parameters except for *CL*_*tot*_*/F*. The calculated bioavailability (*F*) of VCM in the control and PN rats was approximately 0.5 and 1.3 %, respectively; the *F* of VCN in PN rats was approximately 2.6-fold higher than in control rats.

## Discussion and conclusions

The present study investigated the effect of long-term PN on VCM absorption. In order to more adequately induce intestinal atrophy, the period of PN administration was prolonged by 2 days compared with our previously reported method (Fukushima et al. 2015). The changes in body weight and laboratory test results in the present PN rats were similar to those previously reported, except for no obvious hepatic and renal impairment (Additional file 1: Table S1). Therefore, although no visual inspection of the intestines was performed in the present study, intestinal atrophy was very likely induced in the PN rats of the present study.

After intravenous administration, there were no significant differences in VCM pharmacokinetic parameters, including *AUC,* between the control and PN rats (Fig. 1; Additional file 1: Table S2). Because most of the VCM after intravenous administration is eliminated from the kidneys non-metabolized, not only kidney function but also the binding rate of VCM to plasma proteins may have significant effects on VCM distribution. However, the present results indicated that the decrease in plasma protein observed in PN rats did not significantly affect the distribution and elimination of VCM, possibly due to the relatively low protein binding rate of VCM, which was reported to be approximately 47 % (Kusama et al. 1998). Additionally, there was no obvious renal impairment observed in PN rats (Additional file 1: Table S1). Therefore, PN administration in the present study did not alter the distribution and elimination of VCM.

After intraduodenal administration, the *C*_*max*_ and *AUC* of VCM were increased in PN rats, and the bioavailability (*F*) of VCM showed a 2.6-fold increase in PN rats (Additional file 1: Table S2). Generally, a hydrophilic drug such as VCM is absorbed from the intestines via a paracellular pathway, which is modulated by tight junctions (Del Vecchio et al. 2012; Prasad et al. 2003). We previously reported the loss of intestinal epithelial integrity with decreases in total wet weight, mucosal protein content, and villous height of the intestines in rats administered PN for 7 days (Fukushima et al. 2015), and Sun et al. (2008) also reported a decrease in expression of tight junction proteins, such as occluding, zonula occludens and claudin, in PN-administered mice. Although the details of the mechanism underlying these observations still remain unclear, growing evidence indicates that long-term PN administration induces the disintegration of tight junctions along with intestinal atrophy (Feng et al. 2012; Yang et al. 2009). In addition, the slight hypoproteinemia and marked hypotriglyceridemia were induced by the present PN regimen (Additional file 1: Table S2), possibly due to the long-term treatment without lipid emulsion. Combined with the results of the present study, the enhanced oral bioavailability of VCM may have been due to the disruption of tight junctions caused by malnutrition.

The enhanced oral bioavailability of VCM by long-term PN administration was statistically, but not clinically, significant; the VCM bioavailability of 1.3 % (Additional file 1: Table S2) in the present PN rats would be negligible in clinical settings. However, it should be noted here that the PN rats in this study were relatively “healthy” and free from any complications such as renal impairment, colitis, and bacterial translocation (BT). Systemic exposure of VCM may be further enhanced with these complications, particularly in BT, in which bacteria and/or its toxins, far larger than VCM, permeate the intestinal mucosa. Nonetheless, the absorption of VCM itself is not necessarily an unfavorable feature in BT, and may possibly be utilized as a tracer for invaded bacteria. Therefore, the present results demonstrated that long-term PN enhances the oral bioavailability of VCM; however, its clinical significance requires further evaluation.

In conclusion, long-term PN administration did not significantly affect the disposition of VCM, but increased VCM oral bioavailability, possibly due to a loss of tight junction integrity along with intestinal atrophy. The enhanced VCM oral bioavailability was statistically, but not clinically, significant. Therefore, while long-term PN administration plays a role in the enhancement of VCM bioavailability, this effect may be negligible without any complications.

## Additional file

Additional file 1:
**Table S1.** Body weight and laboratory test results of control and PN rats. **Table S2.** Pharmacokinetic parameters of VCM after intravenous (5 mg/kg) and intraduodenal (20 mg/kg) administration to control and PN rats
