# Supplementary material for: Enhanced oral bioavailability of vancomycin in rats treated with long-term parenteral nutrition
Source: Springerplus. 2015 Aug 22;4:442. doi: 10.1186/s40064-015-1228-8 (PMC4546118; doi:10.1186/s40064-015-1228-8)
Supplement: Additional file 1: — Table S1. Body weight and laboratory test results of control and PN rats. Table S2. Pharmacokinetic parameters of VCM after intravenous (5 mg/kg) and intraduodenal (20 mg/kg) administration to control and PN rats [file 40064_2015_1228_MOESM1_ESM.doc]

**Table S1** Body weight and laboratory test results of control and PN rats

| Parameter | (unit) |  | Control | | |  | PN | | |
| --- | --- | --- | --- | --- | --- | --- | --- | --- | --- |
| BW | (g) |  | 253 | ± | 7 |  | 224 | ± | 15^**^ |
| TP | (g/dL) |  | 6.47 | ± | 0.79 |  | 5.37 | ± | 0.38^*^ |
| Alb | (g/dL) |  | 3.95 | ± | 0.21 |  | 3.41 | ± | 0.29^**^ |
| T-Cho | (mg/dL) |  | 53.0 | ± | 5.4 |  | 55.5 | ± | 5.7 |
| TG | (mg/dL) |  | 69.0 | ± | 5.9 |  | 19.6 | ± | 5.6^**^ |
| AST | (IU/L) |  | 116 | ± | 24 |  | 188 | ± | 80 |
| ALT | (IU/L) |  | 65.0 | ± | 21.3 |  | 71.9 | ± | 16.1 |
| BUN | (mg/dL) |  | 22.6 | ± | 6.0 |  | 20.6 | ± | 2.3 |
| CRE | (mg/dL) |  | 0.254 | ± | 0.024 |  | 0.344 | ± | 0.122 |

*BW* body weight, *TP* total protein, *Alb* albumin, *T-Cho* total cholesterol, *TG* triglyceride, *AST* aspartate transaminase, *ALT* alanine transaminase, *BUN* blood urea nitrogen, *CRE* serum creatinine

* *p*<0.05, ***p*<0.01, statistical differences between means were tested using the Mann–Whitney U test

Each value represents the mean ± SD of 8 rats

**Table S2** Pharmacokinetic parameters of VCM after intravenous (5 mg/kg) and intraduodenal (20 mg/kg) administration to control and PN rats

| Parameter | (unit) |  | Control | | |  | | PN | | |
| --- | --- | --- | --- | --- | --- | --- | --- | --- | --- | --- |
| *Intravenous administration, 5 mg/kg* | | | | | | | | | | |
| V_c_ | (mL/kg) |  | 214 | ± | 13 | |  | 234 | ± | 33 |
| V_p_ | (mL/kg) |  | 283 | ± | 29 | |  | 223 | ± | 45 |
| CL_tot_ | (mL/min/kg) |  | 6.44 | ± | 0.46 | |  | 6.22 | ± | 0.22 |
| CLD2 | (mL/min/kg) |  | 6.26 | ± | 0.62 | |  | 4.23 | ± | 1.18 |
| t_1/2α_ | (min) |  | 9.72 | ± | 1.03 | |  | 13.72 | ± | 2.80 |
| t_1/2β_ | (min) |  | 75.7 | ± | 5.7 | |  | 77.4 | ± | 8.3 |
| AUC | (min·ug/mL) |  | 788 | ± | 57 | |  | 807 | ± | 29 |
| *Intraduodenal administration, 20 mg/kg* | | | | | | | | | | |
| ka | (1/min) |  | 0.214 | ± | 0.040 | |  | 0.256 | ± | 0.067 |
| V_c_/F | (L/kg) |  | 158 | ± | 25 | |  | 89 | ± | 28 |
| V_p_/F | (L/kg) |  | 107 | ± | 63 | |  | 29 | ± | 16 |
| CL_tot_/F | (L/min/kg) |  | 1.34 | ± | 0.22 | |  | 0.59 | ± | 0.15^*^ |
| CLD2/F | (L/min/kg) |  | 2.05 | ± | 0.99 | |  | 1.36 | ± | 0.87 |
| C_max_ | (ug/mL) |  | 0.111 | ± | 0.024 | |  | 0.267 | ± | 0.093 |
| T_max_ | (min) |  | 14.3 | ± | 1.2 | |  | 14.0 | ± | 1.7 |
| t_1/2α_ | (min) |  | 31.4 | ± | 12.8 | |  | 26.2 | ± | 12.4 |
| t_1/2β_ | (min) |  | 174 | ± | 50 | |  | 150 | ± | 18 |
| AUC | (min·ug/mL) |  | 15.9 | ± | 2.1 | |  | 41.8 | ± | 10.2^*^ |
|  |  |  |  |  |  | |  |  |  |  |
| F | (%) |  | 0.504 | ± | 0.065 | |  | 1.295 | ± | 0.315^*^ |

*V_c_* distribution volume of the central compartment, *V_p_* distribution volume of the peripheral compartment, *CL_tot_* total body clearance, *CLD2* distribution clearance, *t_1/2α_* distribution half-life, *t_1/2β_* elimination half-life, *AUC* area under the plasma concentration vs time curve, *ka* absorption rate constant, *C_max_* maximum concentration, *T_max_* time to maximum concentration, *F* bioavailability

* *p*<0.05 statistical differences between means were tested using the Mann–Whitney U test

Each value represents the mean ± SE of 4 rats
